# A Short, Valid, and Flexible Web-Based Screener for Mild Cognitive Impairment

**DOI:** 10.1093/geroni/igaa057.871

**Published:** 2020-12-16

**Authors:** Nicole Schmidt, Laura Germine, Eric Grodsky, Chandra Muller, John Robert Warren, Jennifer Manly

**Affiliations:** 1 University of Minnesota, Minneapolis, Minnesota, United States; 2 Harvard Medical School, Belmont, Massachusetts, United States; 3 University of Wisconsin-Madison, Madison, Wisconsin, United States; 4 University of Texas at Austin, Austin, Texas, United States; 5 Columbia University, New York, New York, United States

## Abstract

Clinical assessments for identifying mild cognitive impairment (MCI) can be costly and time-consuming. Few screeners for MCI exist that can be implemented quickly and outside of the clinic using flexible, cost-effective methods, such as web-based, mobile device-friendly assessments. Using data from middle-aged, racially and ethnically diverse Offspring Study participants (N=34 with MCI, N=54 without MCI; mean age: 53.9±3.4), we analyzed the sensitivity and specificity of several web- and telephone-based measures to identify MCI, after accounting for age and education. Web assessments included the Verbal Paired Associates (PA) and Visual PA tests. Phone assessments included the Number Series, Letter and Category Fluency, Number Span Forward & Backward, the AD8, and self-reported memory complaints. The discriminant ability of the web-based Visual PA test for MCI (ROC Area = .69) was comparable to phone-based measures, including the Category Fluency (ROC Area = .69), Number Span Forward (ROC Area = .61) and Backward (ROC Area = .67), and Letter Fluency (ROC Area = .68). Visual PA strongly predicted MCI, with a 98% reduction in the odds of MCI for every additional correct answer (OR=0.02), but our results are imprecise (95%CI: .000 to .76). A web-based Visual PA measure appears comparable to phone assessments in detection of MCI, although substantial uncertainty in its diagnostic precision remains. However, it is short, easily administered on a large-scale, and our evidence suggests that it can provide a sensitive and specific test to refer racially and ethnically diverse individuals for more thorough clinical assessment.

